# Financial burden of prostate cancer in the Iranian population: a cost of illness and financial risk protection analysis

**DOI:** 10.1186/s12962-023-00493-1

**Published:** 2023-11-06

**Authors:** Farbod Alinezhad, Farhad Khalili, Hossein Zare, Chunling Lu, Zahra Mahmoudi, Mahmood Yousefi

**Affiliations:** 1 Bouvé College of Health Sciences, Northeastern University, Boston, MA USA; 2 Management Development, Resources and Planning, Guilan University of Medical Sciences, Rasht, Guilan Iran; 3 Department of Health Policy and Management, Johns Hopkins Bloomberg School of Public Health, Baltimore, USA; 4 Division of Global Health Equity, Brigham & Women’s Hospital, Boston, USA; 5 Department of Global Health & Social Medicine, Harvard Medical School, Boston, USA; 6 Department of Health Economics, School of Management and Medical Informatics, Tabriz University of Medical Sciences, Tabriz, Iran; 7 Tabriz Health Services Management Research Center, Department of Health Economics, School of Management and Medical Informatics, Tabriz University of Medical Sciences., Tabriz, Iran

**Keywords:** Prostate cancer, Cost of illness, Prevalence-based, Health expenditures

## Abstract

**Background:**

Prostate cancer is the second most common cancer in males worldwide and the third most common among Iran’s male population. However, there is a lack of evidence regarding its direct and indirect costs in low and middle-income countries. This study intends to bridge the gap using a cost of illness approach, assessing the costs of prostate cancer from the perspectives of patients, society, and the insurance system.

**Methods:**

Two hundred ninety seven patients were included in the study. Data for a 2-month period were obtained from patients registered at two hospitals (Tabriz, Tehran) in Iran in 2017. We applied a prevalence-based, bottom-up approach to assess the costs of the illness. We used the World Health Organization methods to measure the prevalence and investigate the determinants of catastrophic and impoverishing health expenditures.

**Results:**

We determined the total costs of the disease for the patients to be IRR 68 million (PPP $ 5,244.44). Total costs of the disease from the perspective of the society amounted to IRR 700,000 million (PPP $ 54 million). Insurance companies expended IRR 20 million (PPP $ 1,558.80) per patient. Our findings show that 31% of the patients incurred catastrophic health expenditure due to the disease. Five point forty-four percent (5.44%) of the patients were impoverished due to the costs of this cancer.

**Conclusion:**

We found an alarmingly high prevalence of catastrophic health expenditures among prostate cancer patients. In addition, prostate cancer puts a substantial burden on both the patients and society.

**Supplementary Information:**

The online version contains supplementary material available at 10.1186/s12962-023-00493-1.

## Introduction

Prostate cancer is the second most common cancer in males worldwide. It is estimated that 1.4 million new prostate cancer cases were detected during 2020 [1, 2]. The cumulative risk for a man to have prostate cancer until 75 years of age is 3.73%. Estimations show mortality associated with prostate cancer to be 359,000 in a year; 0.6% of men are losing their lives due to prostate cancer [3]. Prostate cancer is the third most common cancer in Iran [4], with an incidence of 8,937 cases in 2020. Its prevalence is estimated to increase substantially through 2040 to reach 15,798 cases [2, 3].

Complex interrelations between socio-economic and demographic factors are known to cause shifts in disease incidences from communicable to non-communicable diseases [5, 6]. This transition is profound in developing countries and poses a challenge for the health systems in these countries [7–9]. Urbanization of lifestyle and diet may also play a role in the transition to non-communicable diseases in low- and middle-income countries (LMICs) [10]. More than 33% of deaths and 50% of incident cases of the cancers occur in LMICs' [11]. Cancer is the third cause of death in Iran after cardiac diseases and traffic accidents. It is estimated that yearly mortality due to cancer is 30 thousand, and more than 70 thousand new cases of cancer are being diagnosed in the country each year [12, 13].

Cost of illness studies are prevalent in the literature. In the earlier days, these studies were used for the advocacy purpose of resource allocation for the diseases of interest. More widespread use of these studies has been shown to be beneficial in providing a baseline for future interventions, health benefits packages, and defining future research priorities in addition to helping in advocacy efforts [14]. These studies are also useful in cost-effectiveness and cost–benefit enquiries, as they can be used to estimate the return on investment and savings resulting from health-related interventions.

Roehrborn and colleagues have estimated prostate cancer's economic burden in different countries in their review article in 2011. They estimated the annual cancer's direct costs adjusted to 2010 levels as $136 million for England and Wales, $178 million in Australia, $182 million in Netherlands, and $11 Billion in the US [15]. Another major issue posed by costs related to prostate cancer is the possibility of impoverishment and catastrophic health expenditure (CHE) due to these costs. The cancer patients, already incapacitated from their disease and treatments, are at an increased risk of income loss. In combination with the high costs of treatment, this income loss may push many households below the poverty line. Catastrophic health payment has been defined in many ways in previous studies, based on either total expenditure or non-food expenditure of a household. If a household faces catastrophic payments, they lose their capacity to pay for life necessities.

There is a lack of reliable evidence and studies to determine the cost of illness of prostate cancer in low- and middle-income countries, especially the studies which consider both direct and indirect healthcare costs. This study's results may be useful in helping policymakers in Iran and other LMICS to better understand the extent of costs related to this disease and encourage them towards planning an efficient and equitable healthcare system.

## Methods and materials

All estimations were based on data from public hospitals and interview questions. These estimates included economic data, lost labor productivity due to illness, and health indicators. In this study we applied the COI method. This method was developed in more detail by Rice et al. This method shows the social burden of the disease as a quantitative (monetary) value, and since it easily calculates the cost of human capital (lost productivity), it is used more today than other methods. We measured direct and indirect costs of prostate cancer, during the 1 year after the diagnosis [16–21]. We estimated the cost of illness from three perspectives: society, the insurer, and the patient. This approach may help health planners at different levels to use the results of this study.

We used a prevalence-based approach to estimate the costs. This approach uses the costs of an illness in a given period, regardless of when the disease occurred. This approached was used since it is known that the incidence-based approach underestimates the costs for cancers with a high survival rate. In cost of illness (COI), the structure of human capital is usually targeted by investment based on formal and informal education formulated in the form of skills, knowledge, competence and personal experience. Thus, the individual’s lost productivity related to disease is examined socially [22–25]. A bottom-up approach was used in cost estimation due to its higher precision. This approach consists of two stages: in the first stage, health data are measured and quantified, and in the second stage, the cost per unit of service used in production or consumption for specific medical services as well as health care is determined [14].

The participants in the study were selected from the Ghazi hospital in Tabriz and Labbafinejad Hospital in Tehran, Iran in 2017. Sampling was done consecutively among the patients who came to the Ghazi hospital in Tabriz and Labbafinejad Hospital in Tehran for prostate cancer treatment. The questionnaire was completed during two periods) the first questionnaire was completed in May 2017 and the second questionnaire was completed in August 2017). Questionnaires (direct and indirect medical and non-medical expenses) were completed for patients who received care or referred for follow-up.

### Costing

We divided direct costs (DC) in our study into medical (DMC) and non-medical costs (DNMC). Direct medical costs were defined as the sum of the costs of diagnostic services (DSC) (such as imaging and cystoscopy), laboratory and pathology services (LC), therapy costs (TC) (including radiotherapy, hormone therapy, surgery and medicine), in-patient costs (IPC), rehabilitation costs (RC), out-patient costs (OPC) such as physician visits, emergency costs (EC) and costs of medical devices (MDC). Direct non-medical costs included the costs of transportation (TPC), accommodation (AC), and food (FC) attributable to the disease.

$$DC\, = \,DMC\, + \,DNMC$$

where

$$DMC\, = \,DSC\, + \,LC\, + \,TC\, + \,IPC\, + \,RC\, + \,OPC\, + \,EC\, + \,MDC$$

$$DNMC\, = \,{\text{TPC}}\, + \,{\text{AC}}\, + \,{\text{FC}}$$

Indirect costs included “absenteeism” (AL), defined as the loss of productivity due to an absence from the workforce including early retirement (which also equaled the income loss to the patient and his companion) as calculated by the human capital method and the loss of productivity due to “premature death” (mortality cost) (MC). Only the income loss and mortality costs of patients below the age of 65 were included in the calculation for indirect costs. The income loss for all patients' companions was included in the calculation regardless of the patients' age. The daily wage of each patient and his/her companion was multiplied by hospitalization days to calculate the income loss (Additional file 1: Appendix S1).

$$Absenteeism\,income\,loss\,\left( {AIL} \right)\, = \,Daily\,income\,*\,\left( {Days\,hospitalized\, + \,whole\,days\,taken\,off\,due\,to\,the\,disease\, + \,0.5\,*\,Number\,of\,outpatient\,visits\,\left( {in\,home\,city} \right)} \right)$$

$$AL\, = \,AIL\,for\,patient\,if\,below\,65\, + \,AIL\,for\,companion$$

The productivity loss due to premature mortality was calculated by first subtracting the age of the patient from 65, multiplying it by the yearly income of the patient, and multiplying the resultant number by the 1-year mortality rate for prostate cancer in Iran (19% as found in the earlier studies) [26]. For the patients with missing information regarding their income, we used the minimum wage in the country for the year of the study. We used a discount rate of 5.8%, as found in two studies, to be the discount rate for the Iranian population to calculate the loss across the years [27, 28].

We calculated the costs from the patient's perspective (PPC) by adding up the out-of-pocket direct costs and the income loss to the patient and his companion.

$$PPC\, = \,DC_{OOP} \, + \,AIL$$

The costs from the society's perspective (SPC) were calculated by summing the value of the direct costs, absenteeism, and mortality costs and multiplying the sum by the prevalence of the disease in the country (P) as reported by the Global Cancer Observatory [29].

$$SPC\, = \,P\,*\,(DC_{real} \, + \,AIL\, + \,MC)$$

The cost of illness for the financer (FPC) [i.e. insurance fund] was defined as the difference between the real direct costs (both medical and non-medical) and the amount of money paid for them, out of pocket, by the patient.

$$FPC\, = \,DC_{real} \, - \,DC_{OOP}$$

The bills/invoices given to the patients from different providers (e.g., hospitals, labs), in general, included the real costs (costs of the actual services before insurance) of each service. Where the bills did not include these costs, or the patients could only provide us with the data on the out-of-pocket payments, we calculated a ratio (R) for each subtype of health payment (for example, diagnostic procedure costs and laboratory costs) to calculate the total costs. The ratio was calculated by dividing the total costs to out-of-pocket costs within each category, where both were available and non-zero, for each patient and then taking the mean of the results. We then multiplied the out-of-pocket payments by the ratio to estimate the total costs.

We gathered all cost data in Iranian Rials (IRR). We converted it to the purchasing power parity (PPP) US dollars (US$-2018) based on the conversion factor of 13,061.2 per US$ for the year of the study calculated by the World Bank [30].

### Financial risk protection (Catastrophic and impoverishing health expenditures)

We used the methods proposed by the world health organization (WHO) to calculate the indicators for financial risk protection (catastrophic and impoverishing health expenditures) [31].

According to this standard, catastrophic expenditure was defined as a household spending ≥ 40% of their annual capacity to pay (annual income after the expenditure required for subsistence needs) on healthcare needs [32]. As a brief explanation, we calculated a relative poverty line for Iran using the Iran’s Household Expenditure and Income Survey (HEIS) [33]. Then, we used this poverty line to determine if costs attributable to prostate cancer caused the patient's household to fall below the poverty line. To determine the prevalence of catastrophic health expenditure among the patients’ households, we used thresholds of 40 percent relative to the capacity to pay of each household, calculated as the money available to a household after accounting for its food needs [32]. We also calculated the proportion of the individuals (as the patients and their family members) incurring catastrophic health expenditure using the United Nations Sustainable Development Goals approach (SDGs; indicator 3.8.2), which considers health expenditures above 10% and 25% of total household expenditure or income as catastrophic [34]. The proportion of households already under the poverty line was also determined as the health expenditure due to prostate cancer pushed such households further into poverty. Only direct medical and non-medical costs were used in these calculations. We used a logistic regression model to estimate the determinants of catastrophic and impoverishing health expenditures in our study participants. The variables used in the model were age, marital status, and the household size of the participants. All analyses were performed using the R programming language [35].

## Results

### Demographic characteristics of patients

A total of 297 patients were selected for the study. A summary of patient statistics can be found in Table 1. As a summary, the mean age of the patients was 67.5, the majority of them were married, had at least a high school diploma, and were insured.

**Table 1:** Demographic characteristics

Variable	Description	N	%
Age	Mean (SD)	67.5 (11.4)	NA
Patients below the age of 65	Yes	111	37.4
	No	186	62.6
Household size	Mean (SD)	3.2 (1.6)	NA
Marriage status	Married	275	92.6
	Not married	22	7.4
Education	Illiterate	84	28.3
	Elementary or Middle school	7	2.4
	High school diploma	168	56.6
	University graduate	38	12.8
Health insurance coverage	Uninsured	70	23.6
	Public health insurance	41	13.8
	Social security	181	60.9
	Other	5	1.7

### Healthcare service usage

During the 1 year after the diagnosis, most patients utilized some type of diagnostic (71.7%) and laboratory services (92.6%). Thirty-seven point seven percent (37.7%) of the patients reported the use of therapeutic goods and services. Fifteen point two percent (15.2%) and 28.6% of the patient’s used in-patient and out-patient services, respectively. Rehabilitative services were used by 55.6% of the patients in the period of our study.

### Cost of illness from the perspective of the patients

**Fig. 1:**
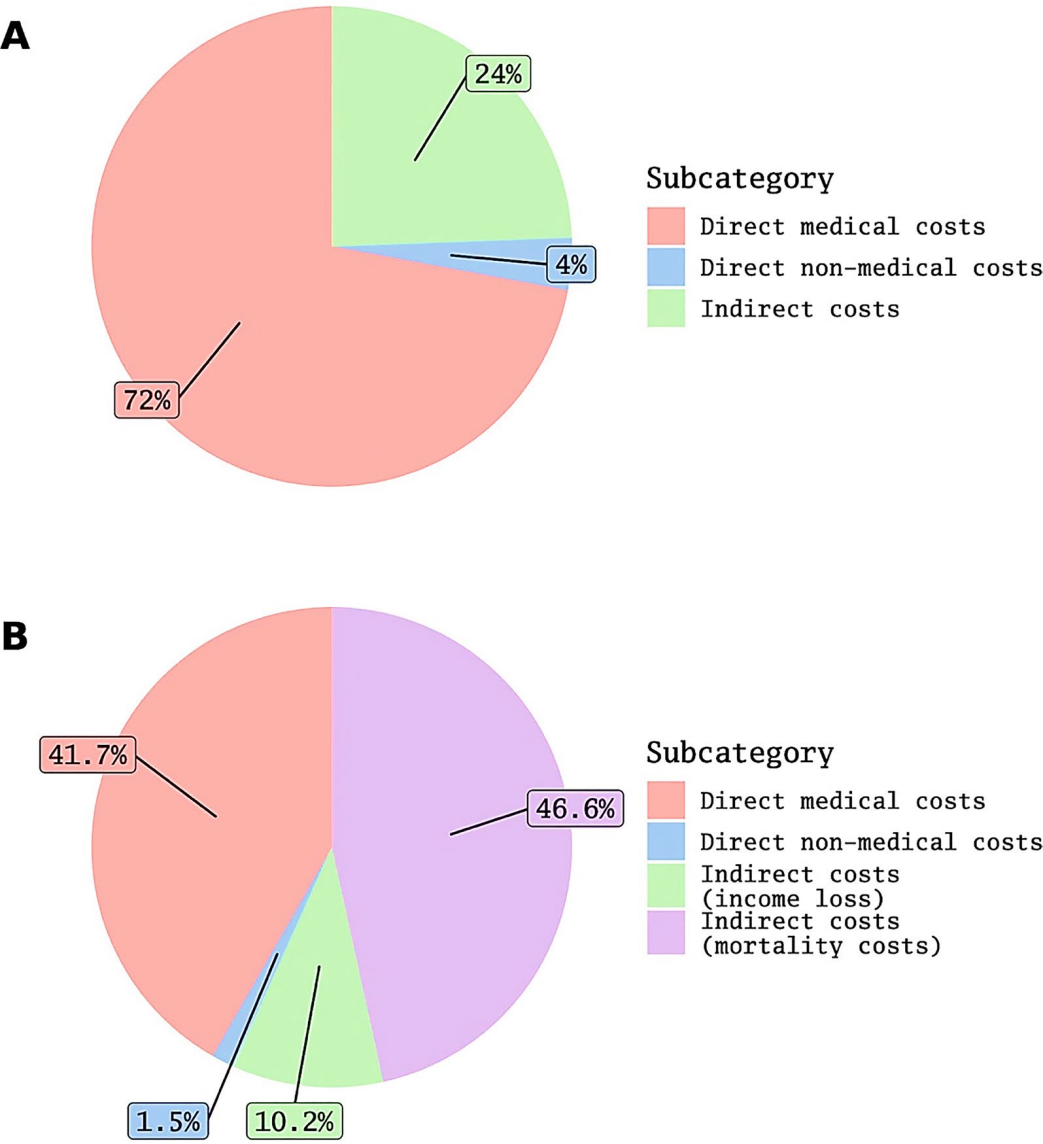
The proportions of the costs attributable to different subcategories from the perspective of the patients A and society B (direct medical costs)

**Fig. 2:**
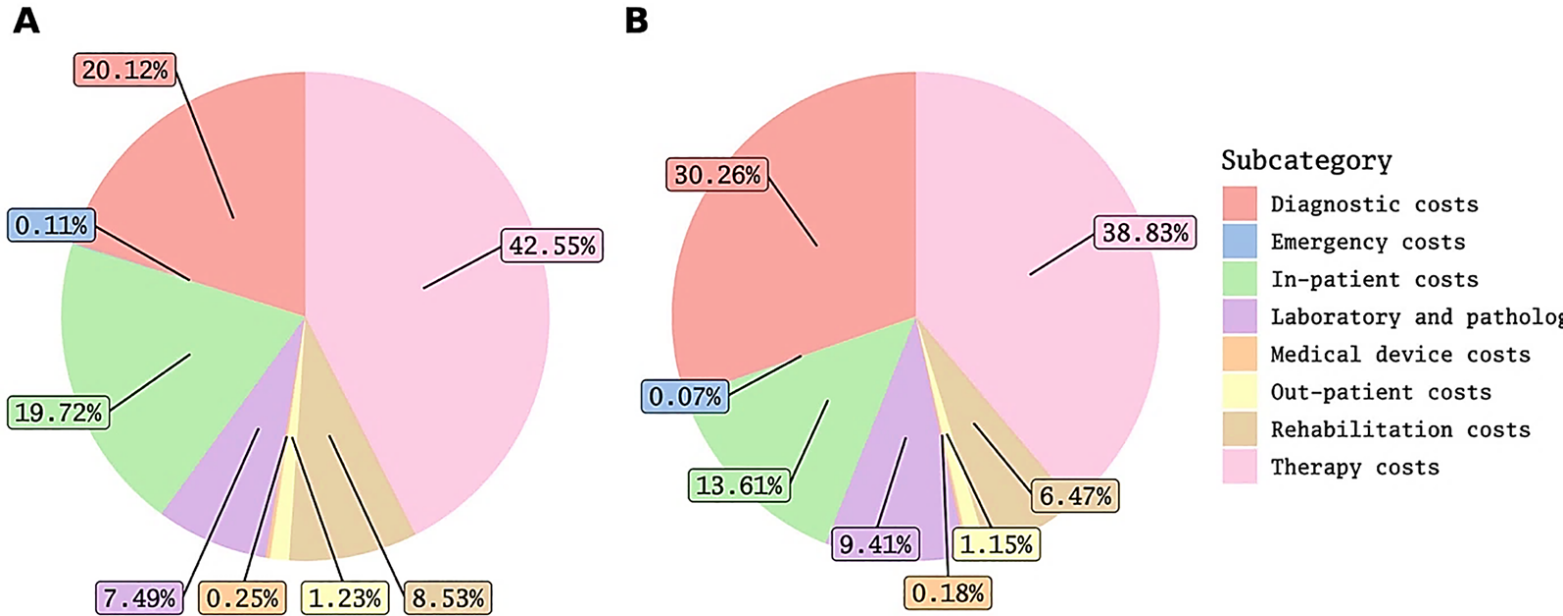
The detailed proportions of the costs attributable to different subcategories from the perspective of the patients A and society B (direct medical costs)

The total costs and costs by category from the patients' perspective are presented in Tables 2 and 3, respectively. Figure 1A summarizes the proportion of costs attributable to each subcategory. Further details on proportions of direct medical costs are available in Fig. 2a.

**Table 2:** The total and category-based costs in Iranian rial (PPP $) from perspective of the patients

	Total costs (million)	Direct costs (million)	Direct medical costs (million)	Direct nonmedical costs (million)	Indirect costs (million)
5th percentile	5.5 (421.1)	1.4 (10.57)	0 (0)	0 (0)	2.2 (168.44)
95th percentile	2.700 (20,511.21)	1.900 (14,231.46)	1.800 (13,909.9)	1.30 (1019.81)	2.90 (2224.91)
Median	2.20 (1667.54)	1.40 (1088.72)	1.20 (900.38)	1.8 (13.78)	6.6 (505.32)
IQR	4.30 (3265.4)	4.40 (3369.52)	3.80 (2871.1)	1.5 (114.84)	6.6 (505.32)
Mean	6.90 (5265.98)	5.20 (3959.03)	4.90 (3772.64)	2.4 (186.4)	1.70 (1306.95)
SD	1.700 (12,776.12)	1.300 (10,200.95)	1.300 (10,192.81)	6.3 (484.69)	9.50 (7272.97)

*IQR* Interquartile range, *SD* Standard deviation

**Table 3:** The total and category-based costs in Iranian rial (PPP $) from the perspective of society calculated using one-year prevalence of the disease

	Total costs (million)	Direct costs (million)	Direct medical costs (million)	Direct nonmedical costs (million)	Indirect costs (million)
5th percentile	40,000 (3.1)	110,000 (0.82)	94,000 (0.72)	0 (0)	93,000 (0.71)
95th percentile	2,400,000 (190)	1,200,000 (88)	1,200,000 (88)	64,000 (4.9)	1,700,000 (130)
Median	300,000 (23)	120,000 (9)	110,000 (8.1)	2,200 (17)	33,000 (2.5)
IQR	830,000 (64)	240,000 (18)	230,000 (18)	6,400 (0.49)	440,000 (34)
Mean	700,000 (54)	300,000 (23)	290,000 (2.)	11,000 (0.83)	400,000 (31)
SD	1,100,000 (82)	620,000 (47)	620,000 (47)	270,000 (21)	850,000 (65)

*IQR* Interquartile range, *SD* Standard deviation

### Cost of illness from the perspective of society

The costs of illness for society (both total and subcategory costs) based on the 1-year prevalence of the disease are presented in Table 3. The average total costs per patient were 700,000 million ($USD PPP 54 million) from the perspective of society. The costs for society per patient, based on the 5-year prevalence of the disease can be found in Additional file 2: Table S1, Additional file 3: Table S2. Figure 1B is a summary of the proportions of costs attributable to each subcategory. A detailed breakdown of direct medical costs for society is available in Fig. 2b.

### Cost of illness from the perspective of the insurance companies

The costs for insurance companies, which only included direct medical costs are available in Table 4.

**Table 4:** The costs in Iranian rial (PPP $) from perspective of the insurance companies

	Costs (million)
5th percentile	0 (0)
95th percentile	68 (5167.98)
Median	8.5 (649.14)
IQR	16 (1258.69)
Mean	20 (1545.66)
SD	42 (3233.15)

*IQR* Interquartile range, *SD* Standard deviation

### Catastrophic and impoverishing health expenditures

Five point forty-four percent (5.44%) of households with prostate cancer patient incurred impoverishing health expenditure in our study. However, a much higher proportion of households suffered from catastrophic health expenditures due to costs attributable to prostate cancer with; 31% of them incurring catastrophic health expenditures using the WHO approach (threshold of 40% of the capacity to pay). In the uninsured subset of the population, the proportion was 37.5%. Using the SDG approach, we found that 35% and 50% of the patients and their family members incurred catastrophic health expenditures using the thresholds of 25% and 10% of the total family expenditure, respectively. The results using 10% and 25% thresholds of family income were 32% and 52% incurring catastrophic health expenditures, respectively.

Eighteen point seven percent (18.7%) of the households were already below the poverty line, getting pushed further into poverty due to the costs of the illness.

### Determinants of catastrophic and impoverishing health expenditures

Out of the variables included in the logistic regression model (mentioned in the material and methods section), only the age of the patient was found to be a significantly negative determinant of the possibility of catastrophic health expenditures with an odds ratio of 0.98 (95% CI 0.96, 0.99) and a P value of 0.04 (Additional file 4: Table S3).

## Discussion

Our study provides a detailed picture of the costs of prostate cancer from three different perspectives, namely, the patient and his family, society, and the healthcare financing system (i.e. insurance providers). We studied different subcategories of direct and indirect costs and described how much of the total healthcare costs each subcategory accounts for.

The high proportion of the uninsured in our study (23.6%) could be one of the major contributors to the high prevalence of catastrophic healthcare expenditure.

From the patients’ perspective, direct medical costs were the major contributor to the high costs experienced by patients, as signified by the high proportion of catastrophic expenditures among them.

Our results regarding the costs from society’s perspective were comparable to the results in other countries. We calculated the median per-patient direct medical costs to be 5,245$, which was substantially lower than the figures for the USA, Canada, and Italy, but similar to the results for UK, Germany, France, Italy, Spain [15, 36–38]. These differences can be attributed to the variations in the screening and treatment patterns between these countries and Iran, as the USA’s treatments are known to be more aggressive in comparison to the rest of the world [15]. Our results for direct costs were higher in comparison to another study performed in Iran. We are, however, unable to make a clear comparison between our and their study, as there were differences in the methodologies, as well as uncertainties regarding their use of currencies (as they did not include a clear description of the method they used to convert Iranian currency to USD and if they used PPP) [39].

We found a relatively even distribution of costs between direct and indirect subcategories for society. This finding was consistent with the earlier findings in the US [40]. The major contributor to the indirect costs was the mortality cost associated with the premature death of the patients. This finding may also be attributable to our study’s human capital approach, which is known to overestimate such costs in comparison to the friction cost approach [41]. For both society and patients, the major contributors to direct medical costs were therapeutic, diagnostic, and in-patient costs. This was in line with the results for other cancers in Iran as well as the results for prostate cancer in other countries [37–42].

We found an alarmingly high prevalence of catastrophic healthcare expenditures due to prostate cancer in the country. This finding was consistent across different methods and thresholds of calculating such expenditures. This signifies a need for much better coverage of the costs related to this cancer in the Iranian population, especially regarding the significant sources of expenditure for the patients, including drugs, diagnostic services, and in-patient care.

The importance of age in determining the probability of financial catastrophe for the patients can be a result of the effects of the increased costs of illness due to a loss of income in younger patients, as well as the use of costlier treatment options for these patients.

Our study has limitations resulting from its bottom-up approach. Misreporting of certain costs and income may occur with this approach. Such an approach, however, is still valuable, as it enables one to gain valuable information directly from the patient, which is not possible when using a top-down approach using, for example, with administrative data. Also, the methodology leads to an issue of the higher likelihood of missing values, which we tried to address by using the available data from the other patients. Furthermore, the patients that were interviewed were being treated in a hospital in a metropolitan area, which might lead to generalizability issues regarding the patients that might have had access issues to such a hospital, such as the rural patients.

## Conclusion

In conclusion, we have found that the Iranian society, its healthcare financers, and prostate cancer patients incur substantial costs due to prostate cancer. The similarities between our results and the results worldwide show that the country’s situation regarding the effective use of resources is better than in some countries but in need of further improvements. In this time of financial instability in Iran, it is essential to move towards more informed decision making in the healthcare industry to increase the sustainability of healthcare services. Strategic purchasing and performance-based payment systems may be options in this regard. Such approaches combined with the efforts to improve the coverage of different costs associated with prostate cancer may help patients and families to not incur catastrophic costs and impoverishment.

### Supplementary Information

**Additional file 1: Appendix S1.** Examples of costs related to COI.

**Additional file 2: Table S1.** The total and category-based costs in Iranian rial (PPP $) per patient from perspective of the society (IQR: Interquartile range, SD: Standard deviation).

**Additional file 3: Table S2.** The total and category-based costs in Iranian rial (PPP $) from perspective of the society (calculated using five-year prevalence of the disease) (IQR: Interquartile range, SD: Standard deviation).

**Additional file 4: Table S3.** Determinants of catastrophic and impoverishing health expenditures.

## Data Availability

The data used in this study are access on request from the corresponding author.

## Abbreviations

COI: Cost of illness
LMICs: Low- and middle-income countries
CHE: Catastrophic health expenditure
DC: Direct costs
DMC: Direct medical costs
DSC: Diagnostic services costs
LC: Laboratory and pathology services costs
TC: Therapy costs
IPC: In-patient costs
RC: Rehabilitation costs
OPC: Out-patient costs
EC: Emergency costs
MDC: Medical devices costs
DNMC: Direct non-medical costs
TPC: Transportation costs
AC: Accommodation costs
FC: Food costs
MC: Mortality cost
PPC: Patient’s perspective cost
SPC: Society’s perspective cost
PPP: Purchasing power parity
IQR: Interquartile ranges
SE: Standard errors
