# Supplementary material for: Financial burden of prostate cancer in the Iranian population: a cost of illness and financial risk protection analysis
Source: Cost Eff Resour Alloc. 2023 Nov 6;21:84. doi: 10.1186/s12962-023-00493-1 (PMC10629147; doi:10.1186/s12962-023-00493-1)
Supplement: Supplementary file 1 — Additional file 1: Appendix S1. Examples of costs related to COI. [file 12962_2023_493_MOESM1_ESM.docx]

Appendix1. Examples of costs related to COI

| \| Direct costs in health care \| \| --- \| | | \| Direct costs in non-health care \| \| --- \| | \| Indirect costs \| \| --- \| |
| --- | --- | --- | --- | --- | --- | --- |
| \| - Institutional inpatient care  Hospitalization specialized unit (ICU, CCU)  Nursing home  Terminal care or Hospice  - Institutional outpatient service  Clinic and ER  - Home health care  - Physician services  General practitioner (GPs)  Specialists  - Ancillary services  Nurses (RNs, Nursing Aid)  Nutritionists  Physical therapist  Ambulance  - Overhead allocated to technology  Fixed costs of utilities  Space and storage  Support services  Capital costs (depreciation)  Construction of facilities  Relocation expenses  Device or equipment cost  - Variable costs of utilities  - Medications (prescription and non-prescription)  Drug costs  Training in new procedures  Dispensing and administration  Monitoring \| \| --- \| | \| - Devices and applications  - D rugs, supplies, devices provided by household  - Diagnostic test  Imaging  Laboratory testing  - Treatment services  Surgery  Consumable supplies, personnel time, equipment  Treatment of complications  Blood products  Oxygen  Radiation therapy  Special diets  - Prevention services  Screening space  Vaccination, prophylaxis  Disease prevention in contacts of known cases  - Rehabilitation  - Training and education  Health education  Self-care training for patients  Life-support skills for general population \| \| --- \| | \| Social services  Counseling  Retraining  - Program evaluation  Monitoring impact of program or technology  Data analysis  - Repair of property destruction  - Legal costs  - Transportation costs  - Time (searching, traveling, waiting etc.)  - Childcare or Housekeeping \| \| --- \| | \| Productivity losses  Morbidity  Mortality  Impairment  Jon absenteeism  - Foregone leisure time  - Time spent by family & visitors attending patient \| \| --- \| |
