# Supplementary material for: Financial burden of prostate cancer in the Iranian population: a cost of illness and financial risk protection analysis
Source: Cost Eff Resour Alloc. 2023 Nov 6;21:84. doi: 10.1186/s12962-023-00493-1 (PMC10629147; doi:10.1186/s12962-023-00493-1)
Supplement: Supplementary file 2 — Additional file 2: Table S1. The total and category-based costs in Iranian rial (PPP $) per patient from perspective of the society (IQR: Interquartile range, SD: Standard deviation). [file 12962_2023_493_MOESM2_ESM.docx]

|  | Total costs ($) | Direct costs | Direct medical costs | Direct nonmedical costs | Indirect costs |
| --- | --- | --- | --- | --- | --- |
| 5^th^ percentile | 9.4E+06 (723.06) | 2.5E+06 (194.5) | 2.2E+06 (170.15) | 0 (0) | 2.2E+06 (168.44) |
| 95^th^ percentile | 5.8E+08 (44028.08) | 2.7E+08 (20795.95) | 2.7E+08 (20795.95) | 1.5E+07 (1148.44) | 4E+08 (30321.7) |
| median | 7E+07 (5385.42) | 2.8E+07 (2113.13) | 2.5E+07 (1913.3) | 5.1E+05 (39.05) | 7.7E+06 (589.54) |
| IQR | 2E+08 (14992.61) | 5.5E+07 (4244.63) | 5.5E+07 (4182.62) | 1.5E+06 (114.84) | 1E+08 (8012.42) |
| mean | 1.7E+08 (12703.05) | 7.2E+07 (5504.69) | 6.9E+07 (5308.92) | 2.6E+06 (195.77) | 9.4E+07 (7198.36) |
| SD | 2.5E+08 (19457) | 1.5E+08 (11158.3) | 1.5E+08 (11152.55) | 6.4E+06 (488.44) | 2E+08 (15436.41) |

Additional table 1. The total and category-based costs in Iranian rial (PPP $) per patient from perspective of the society for the raw data (IQR: Interquartile range, SD: Standard deviation)
