# Supplementary material for: Financial burden of prostate cancer in the Iranian population: a cost of illness and financial risk protection analysis
Source: Cost Eff Resour Alloc. 2023 Nov 6;21:84. doi: 10.1186/s12962-023-00493-1 (PMC10629147; doi:10.1186/s12962-023-00493-1)
Supplement: Supplementary file 3 — Additional file 3: Table S2. The total and category-based costs in Iranian rial (PPP $) from perspective of the society (calculated using five-year prevalence of the disease) (IQR: Interquartile range, SD: Standard deviation). [file 12962_2023_493_MOESM3_ESM.docx]

|  | Total costs | Direct costs | Direct medical costs | Direct nonmedical costs | Indirect costs |
| --- | --- | --- | --- | --- | --- |
| 5^th^ percentile | 1.3E+11 (1E+07) | 3.5E+10 (2.7E+06) | 3.1E+10 (2.4E+06) | 0 ( 0) | 3E+10 (2.3E+06) |
| 95^th^ percentile | 8E+12 (6.1E+08) | 3.8E+12 (2.9E+08) | 3.8E+12 (2.9E+08) | 2.1E+11 (1.6E+07) | 5.5E+12 (4.2E+08) |
| median | 9.7E+11 (7.5E+07) | 3.8E+11 (2.9E+07) | 3.5E+11 (2.6E+07) | 7.1E+09 (5.4E+05) | 1.1E+11 (8.2E+06) |
| IQR | 2.7E+12 (2.1E+08) | 7.7E+11 (5.9E+07) | 7.6E+11 (5.8E+07) | 2.1E+10 (1.6E+06) | 1.4E+12 (1.1E+08) |
| mean | 2.3E+12 (1.8E+08) | 1E+12 (7.6E+07) | 9.6E+11 (7.3E+07) | 3.5E+10 (2.7E+06) | 1.3E+12 (1E+08) |
| SD | 3.5E+12 (2.7E+08) | 2E+12 (1.5E+08) | 2E+12 (1.5E+08) | 8.8E+10 (6.8E+06) | 2.8E+12 (2.1E+08) |

Additional table 2. The total and category-based costs in Iranian rial (PPP $) from perspective of the society for the raw data (calculated using five-year prevalence of the disease) (IQR: Interquartile range, SD: Standard deviation)
