# Supplementary material for: Financial burden of prostate cancer in the Iranian population: a cost of illness and financial risk protection analysis
Source: Cost Eff Resour Alloc. 2023 Nov 6;21:84. doi: 10.1186/s12962-023-00493-1 (PMC10629147; doi:10.1186/s12962-023-00493-1)
Supplement: Supplementary file 4 — Additional file 4: Table S3. Determinants of catastrophic and impoverishing health expenditures. [file 12962_2023_493_MOESM4_ESM.docx]

|  | Catastrophic Expenditures | | | | Impoverishing Expenditures | | | |
| --- | --- | --- | --- | --- | --- | --- | --- | --- |
|  | Odds | Confidence Interval | | P value | Odds | Confidence Interval | | P value |
|  |  | 2.5 % | 97.5 % |  |  | 2.5 % | 97.5 % |  |
| Intercept | 2.00 | 0.36 | 11.30 | 0.43 | 0.89 | 0.04 | 24.19 | 0.95 |
| Age | 0.98 | 0.96 | 1.00 | 0.04 | 0.96 | 0.92 | 1.00 | 0.08 |
| Marriage Status Single vs. Married | 0.94 | 0.33 | 2.41 | 0.89 | 1.23 | 0.15 | 5.60 | 0.82 |
| Household Size | 1.02 | 0.86 | 1.19 | 0.83 | 0.89 | 0.57 | 1.24 | 0.55 |

Additional table 3**.** Determinants of catastrophic and impoverishing health expenditures
